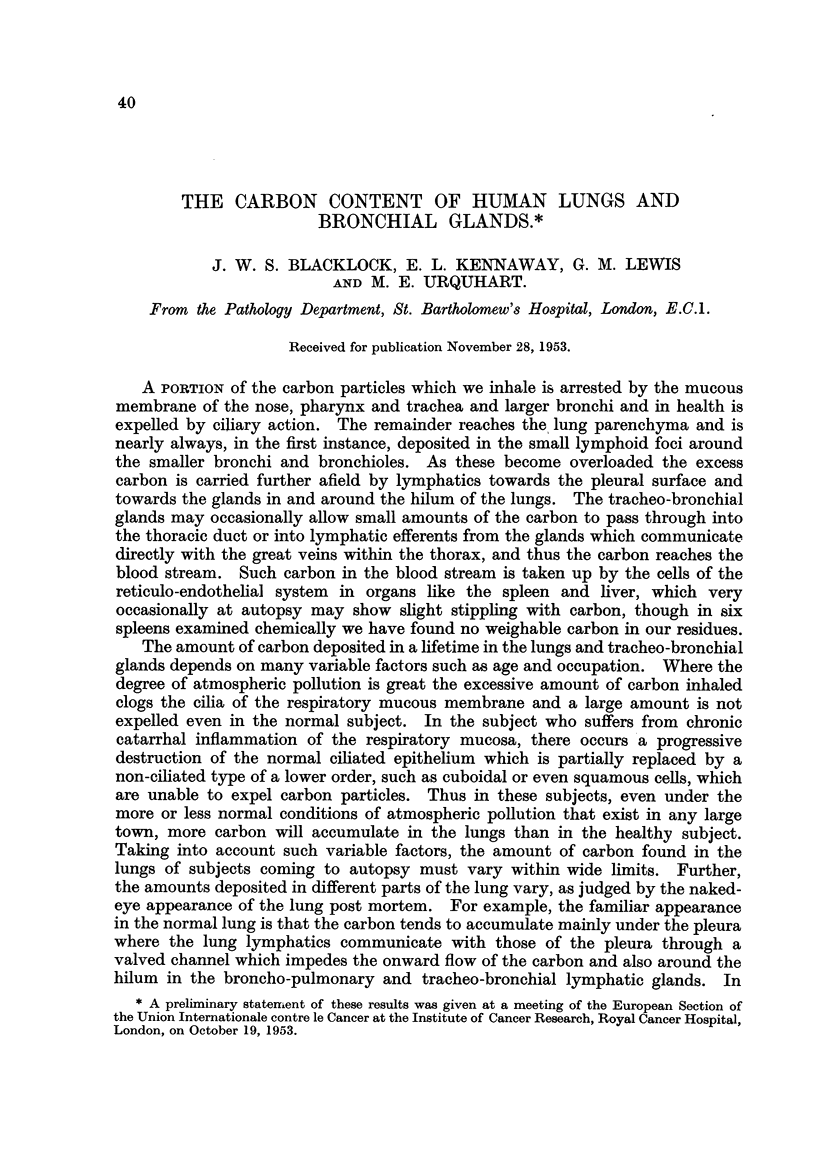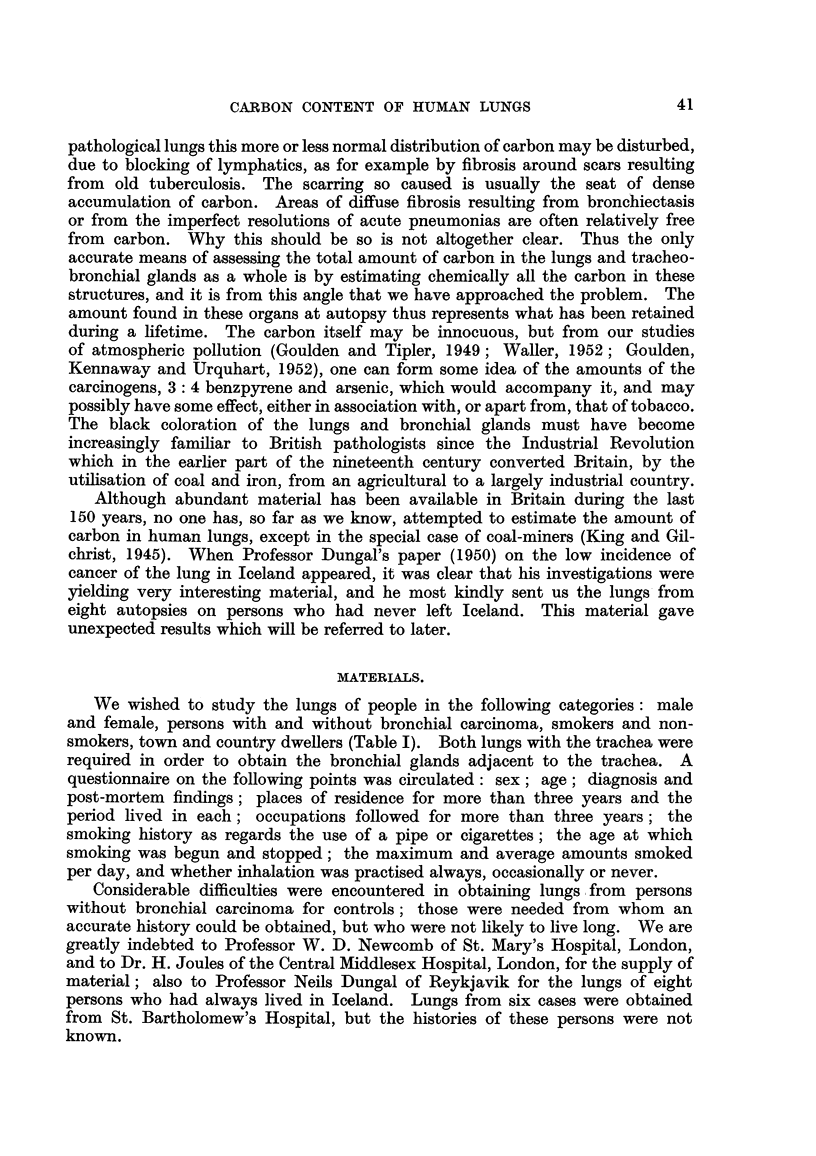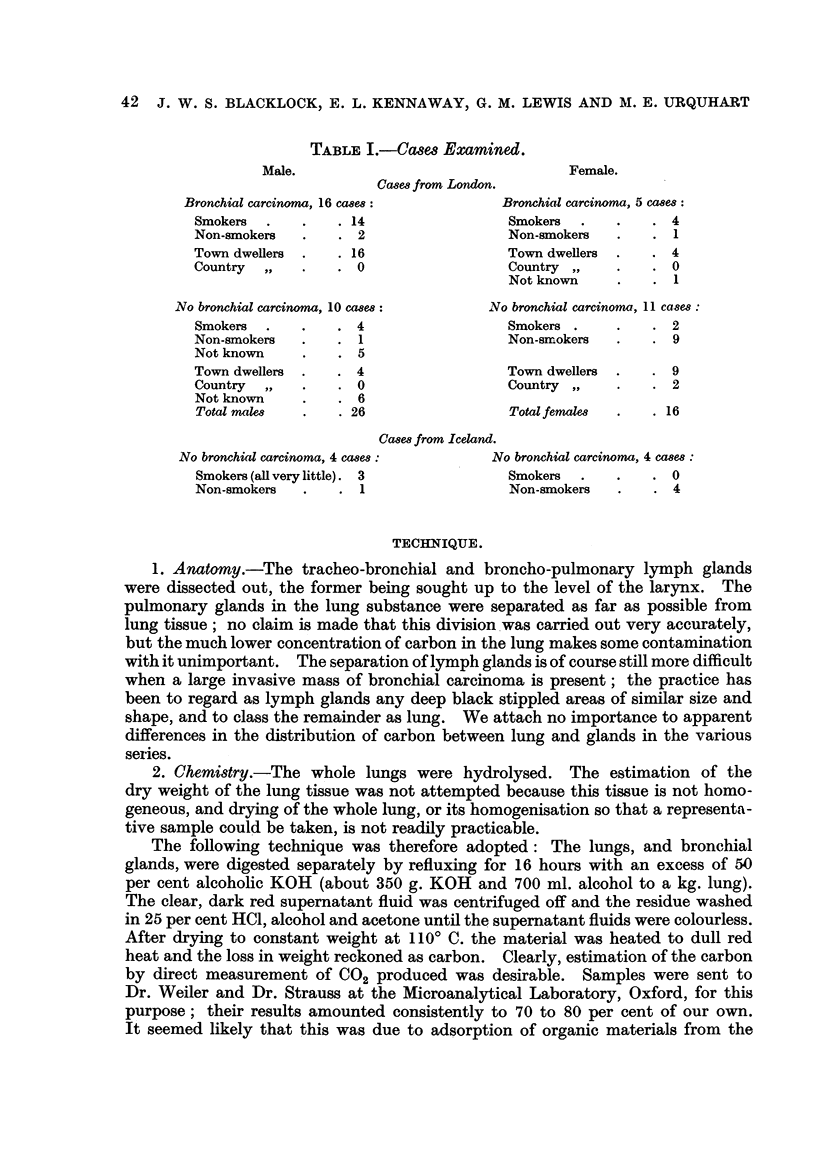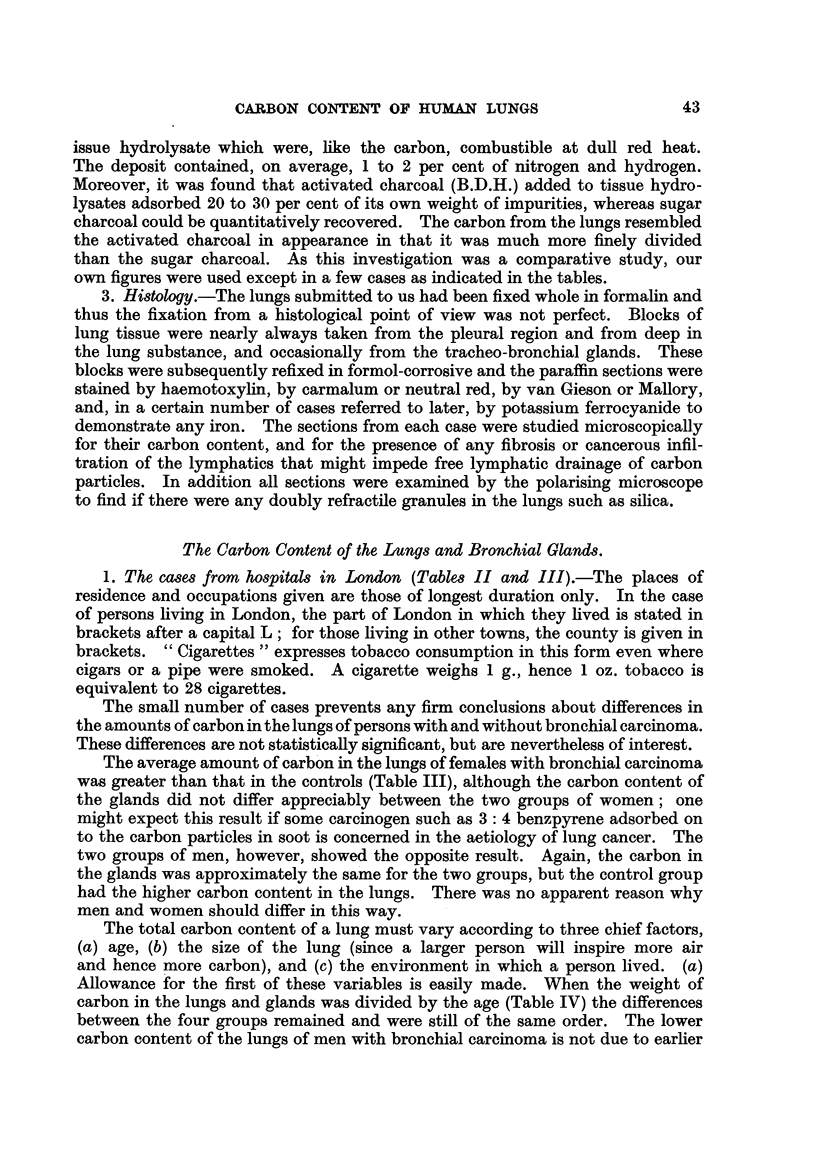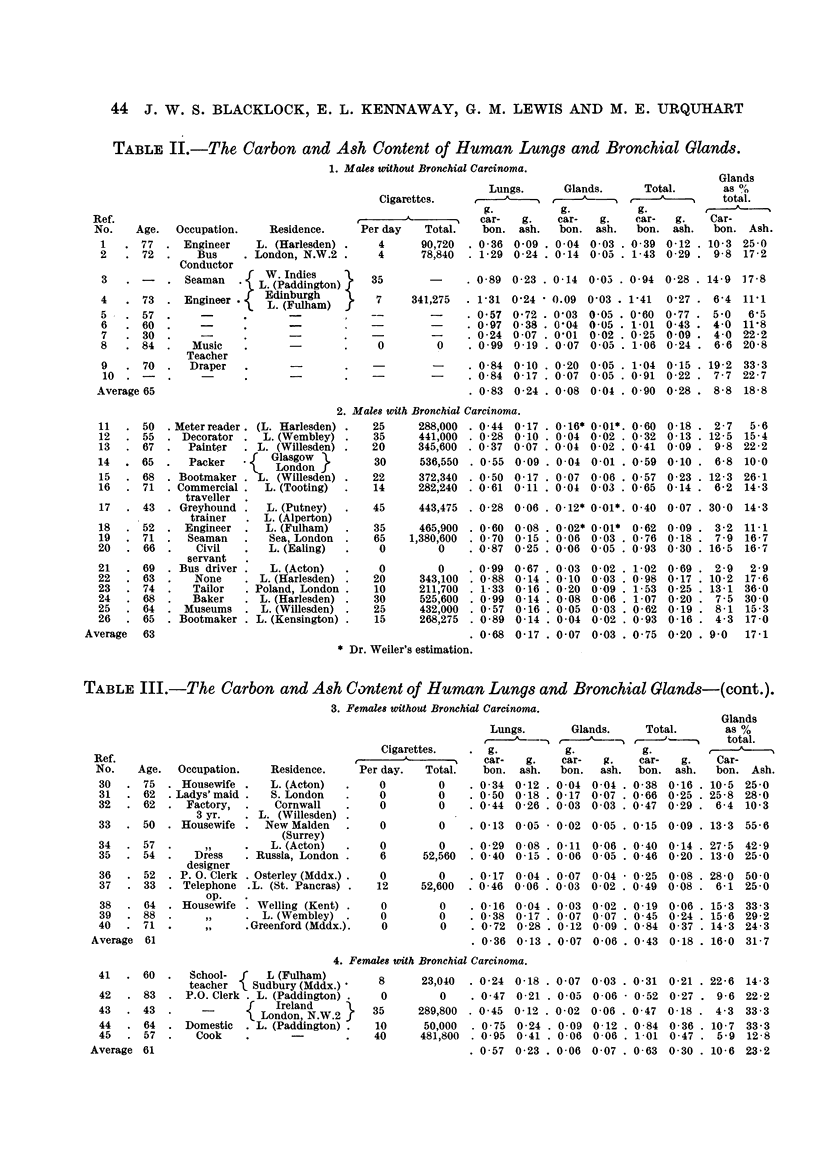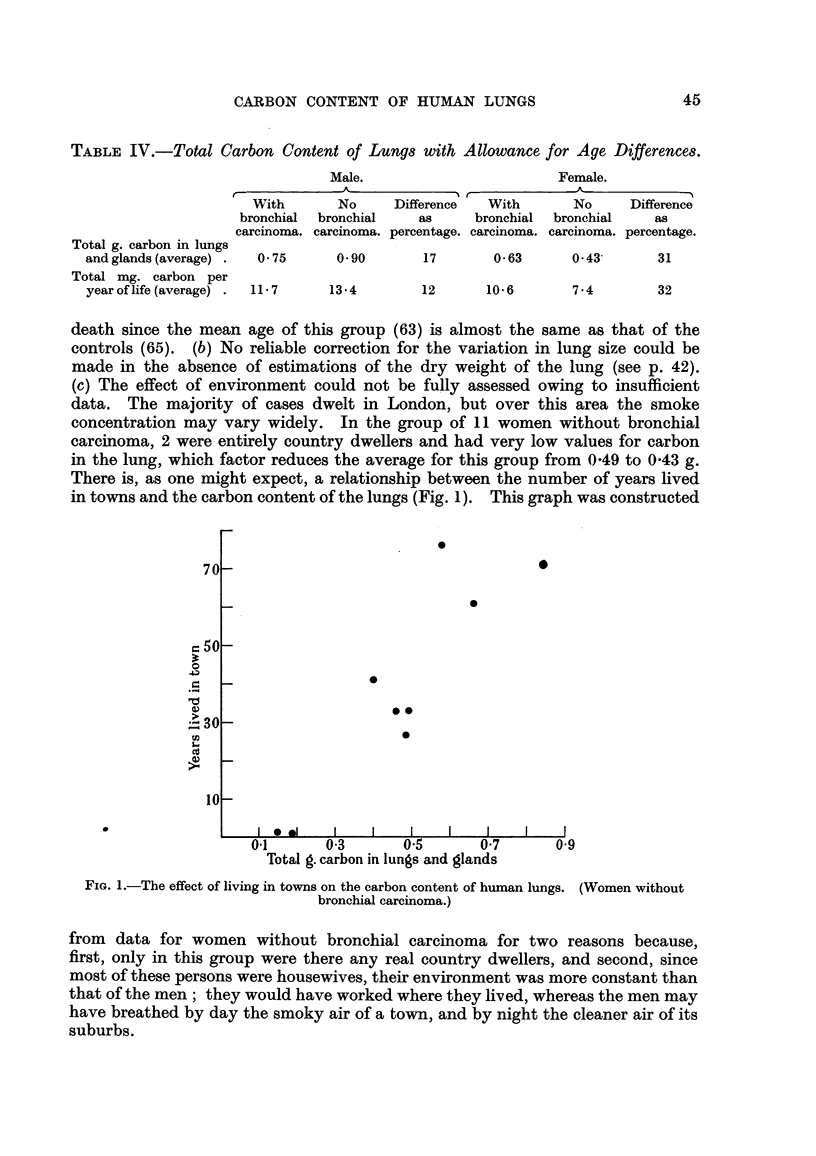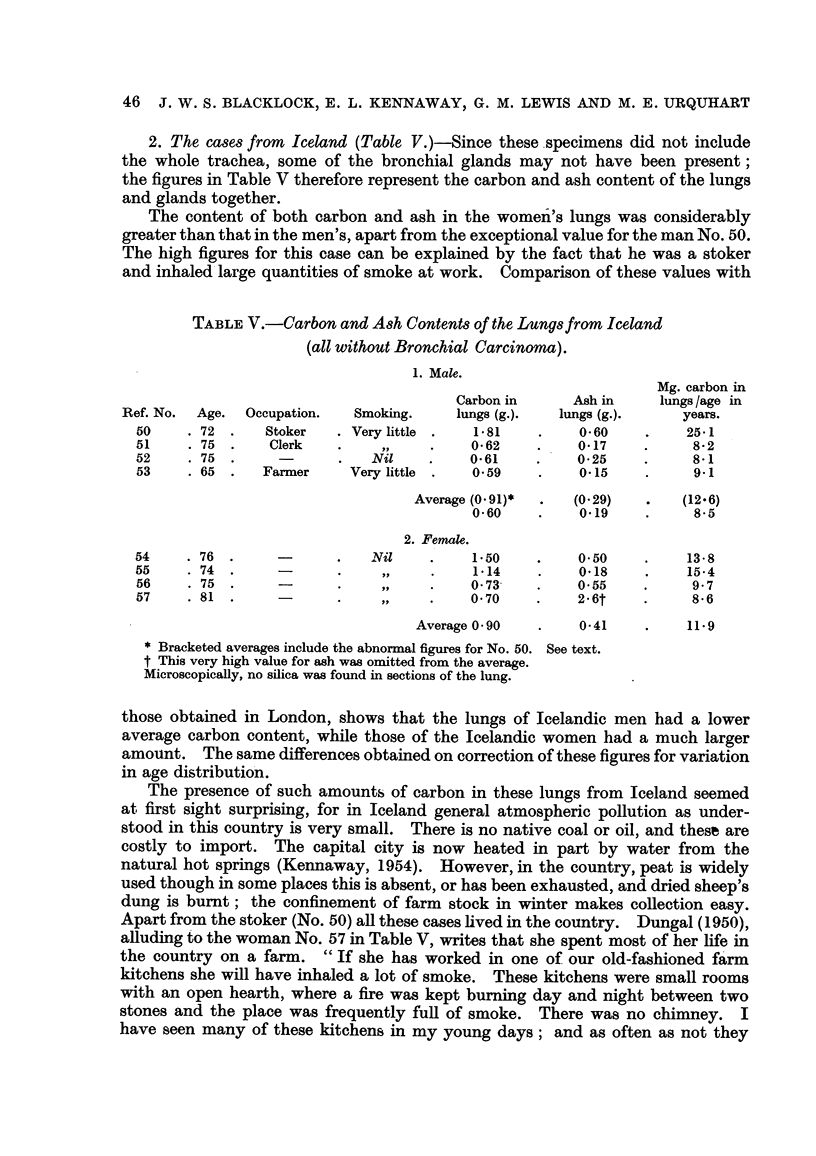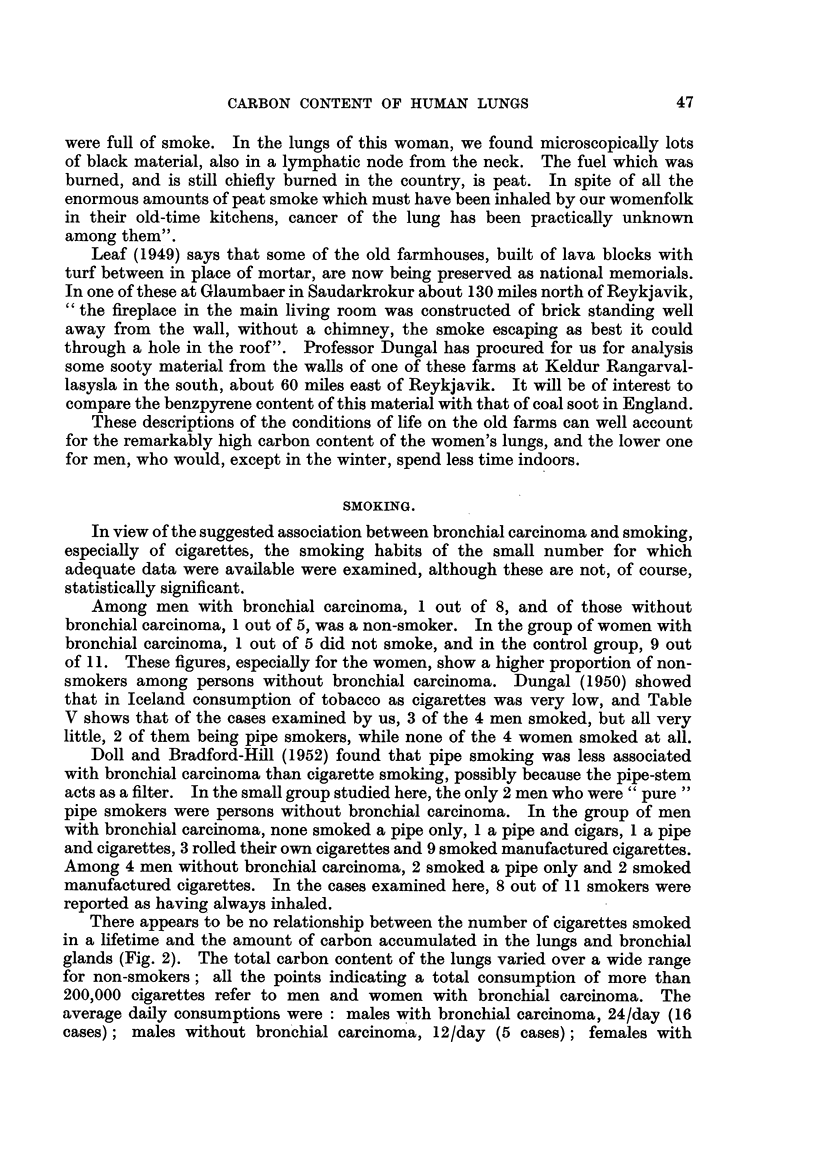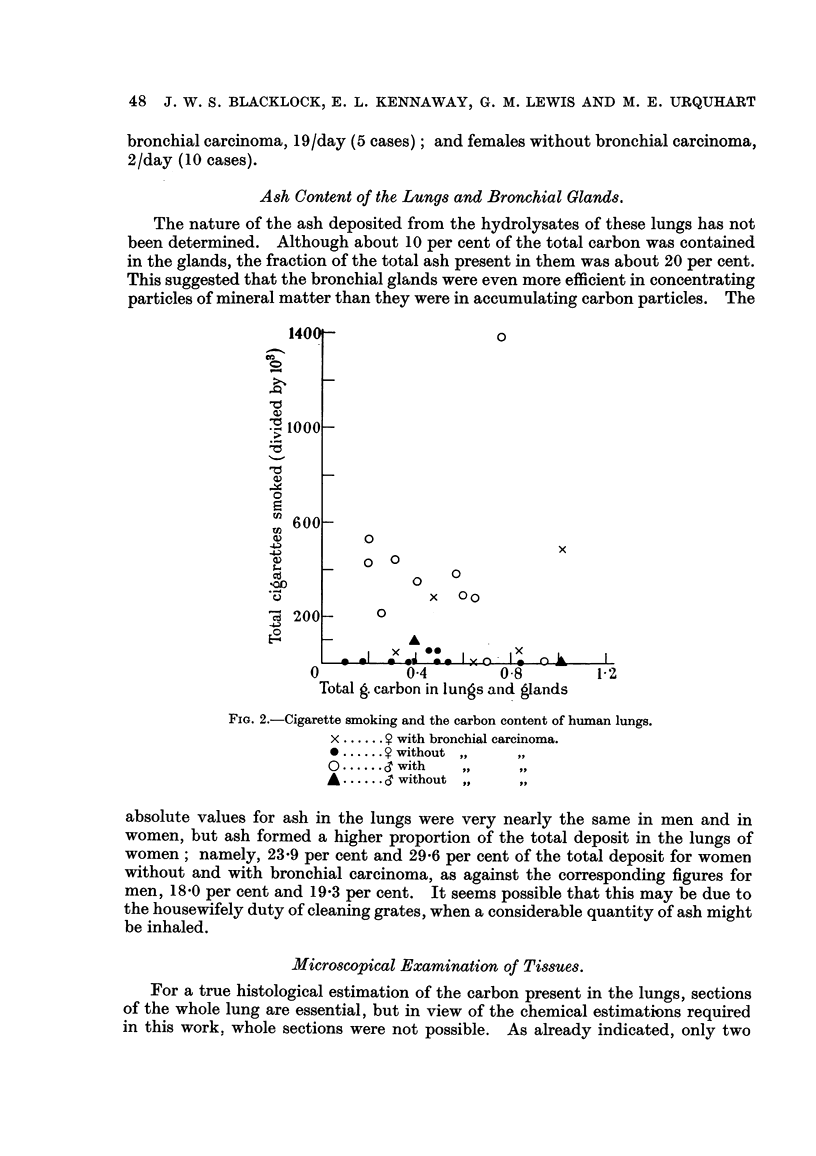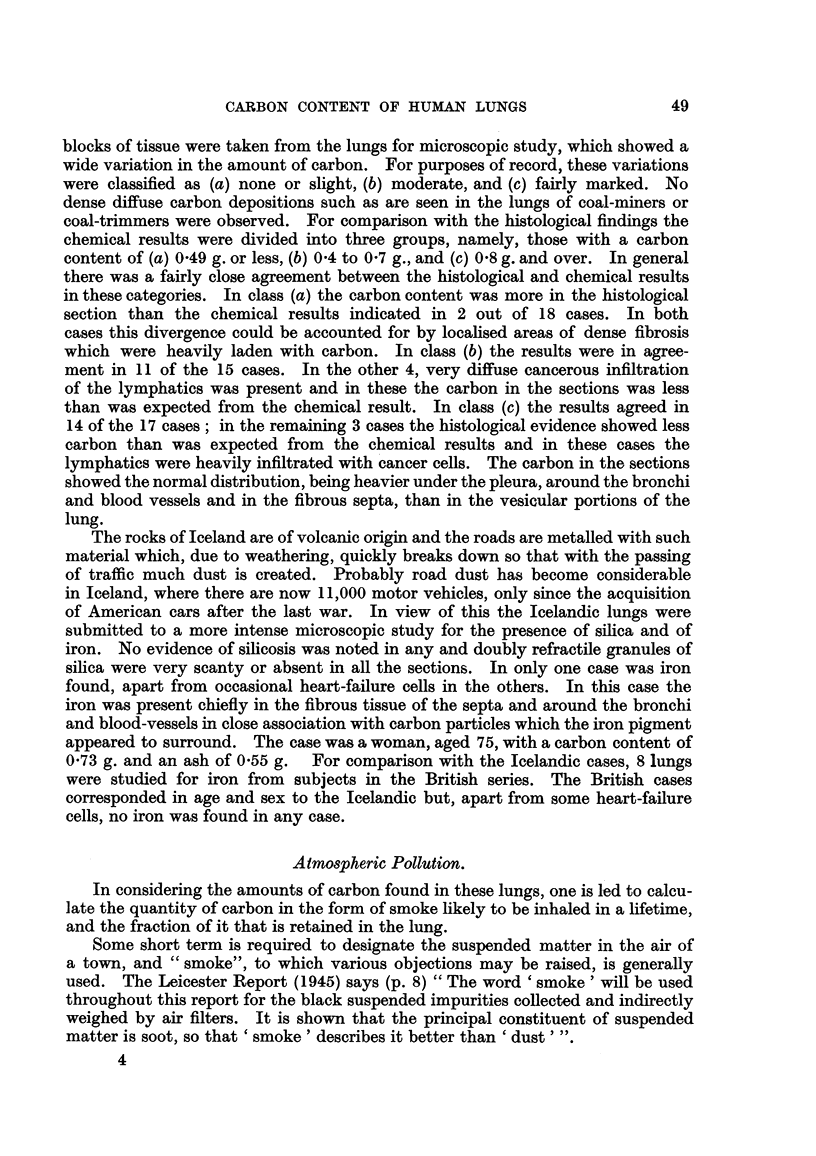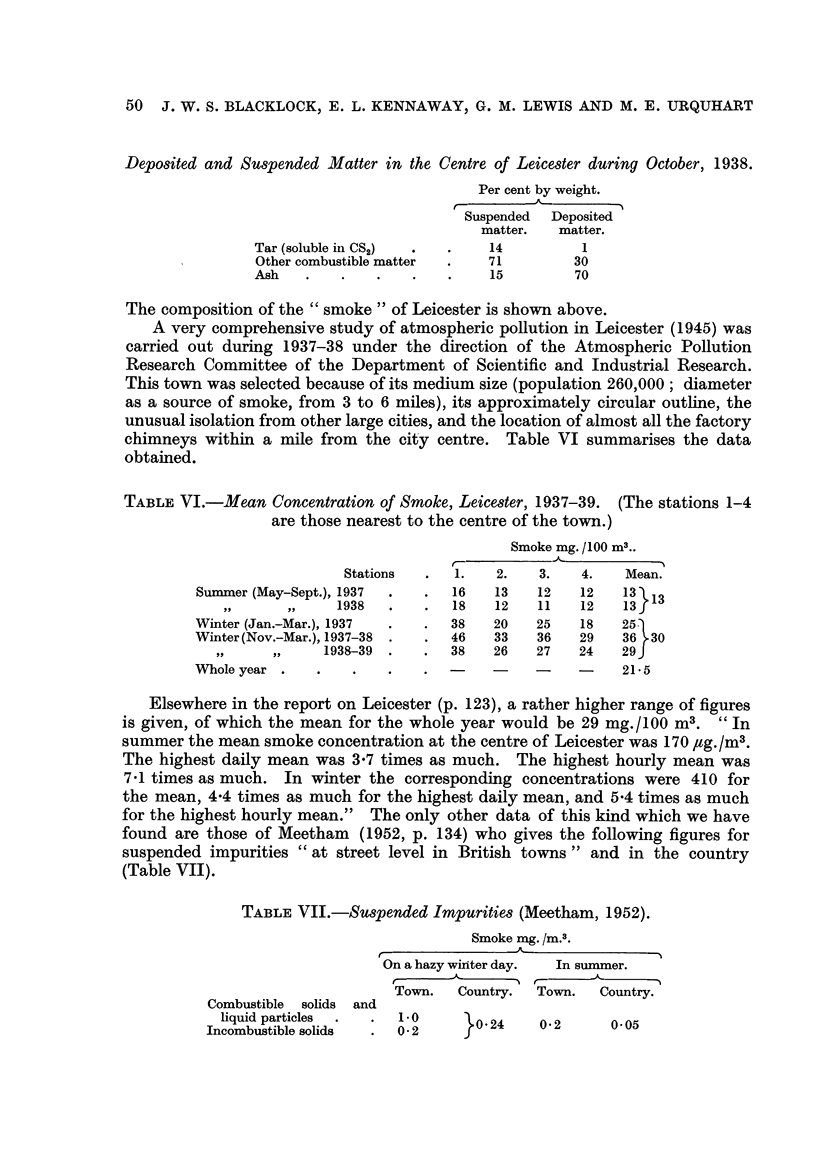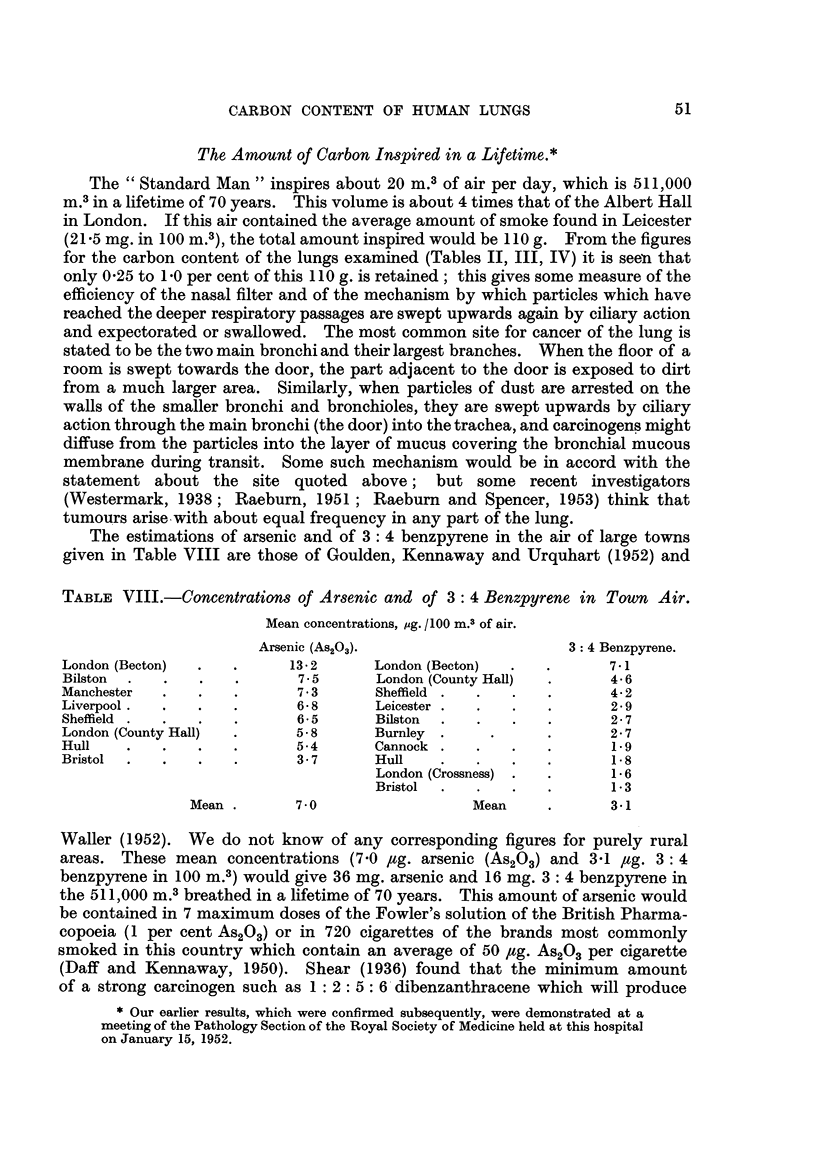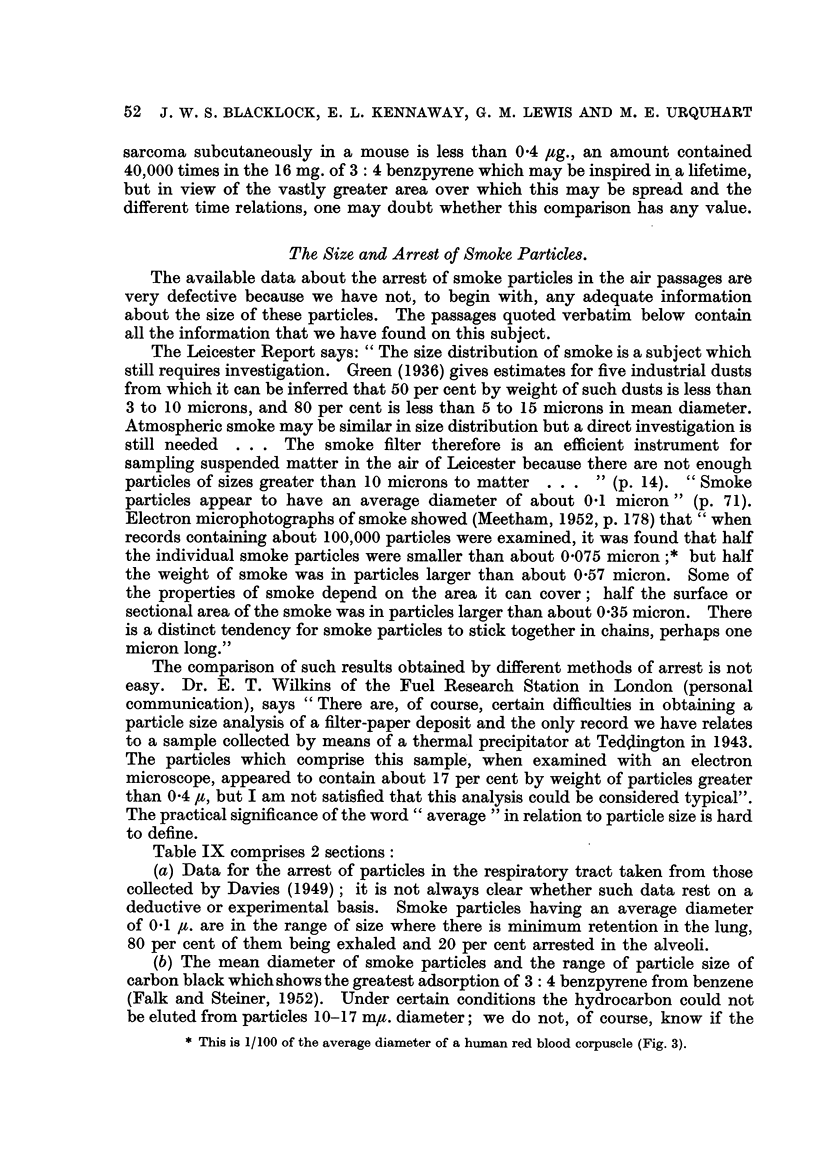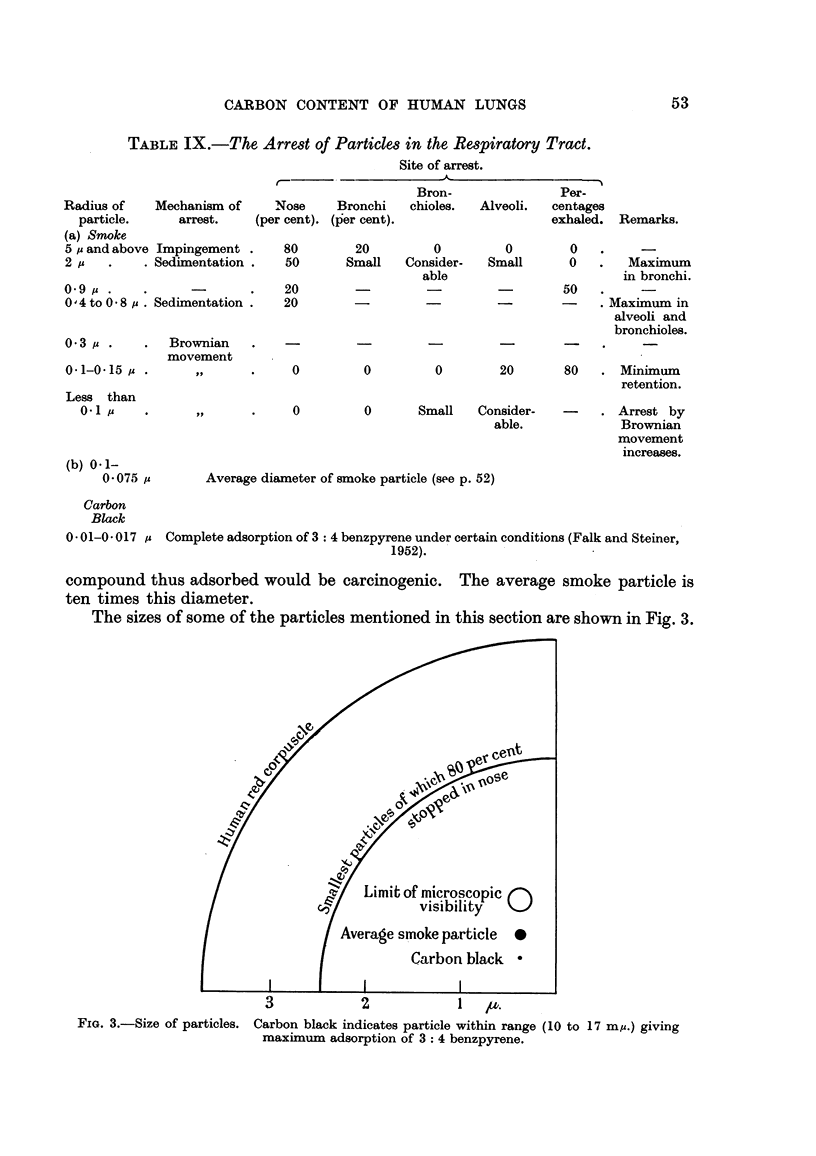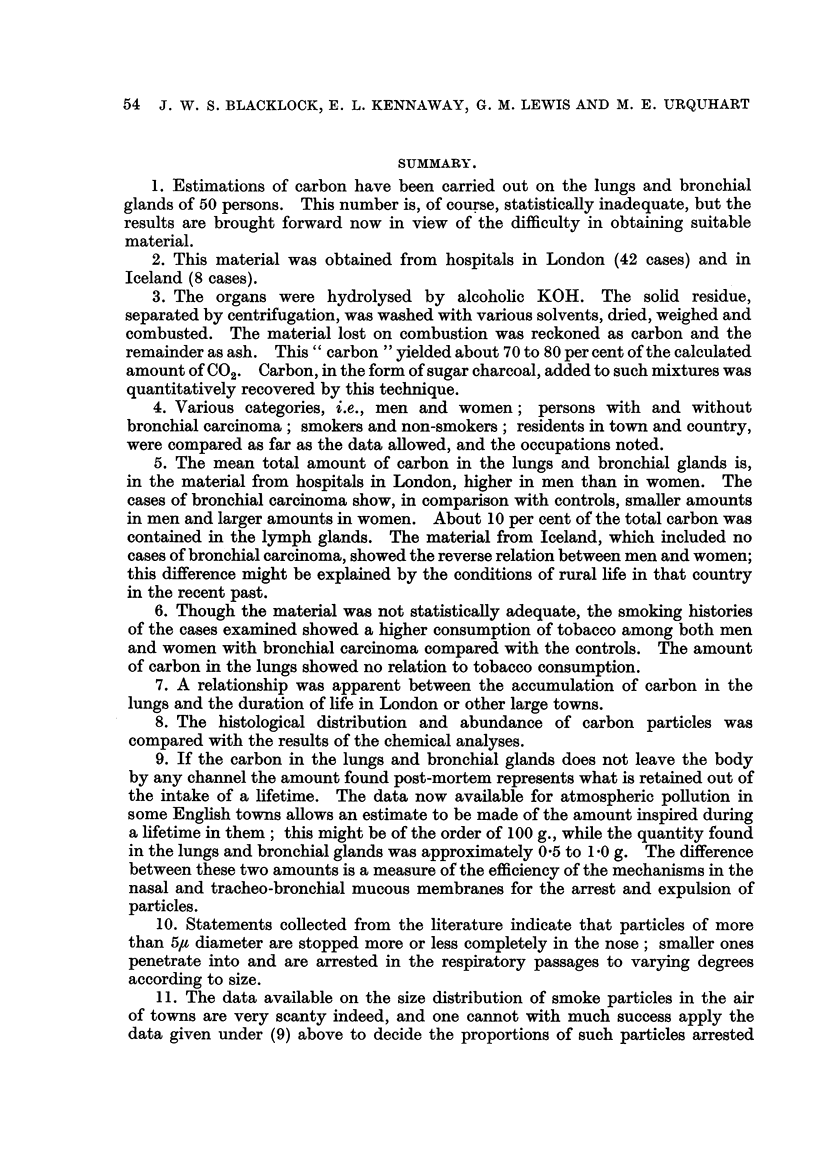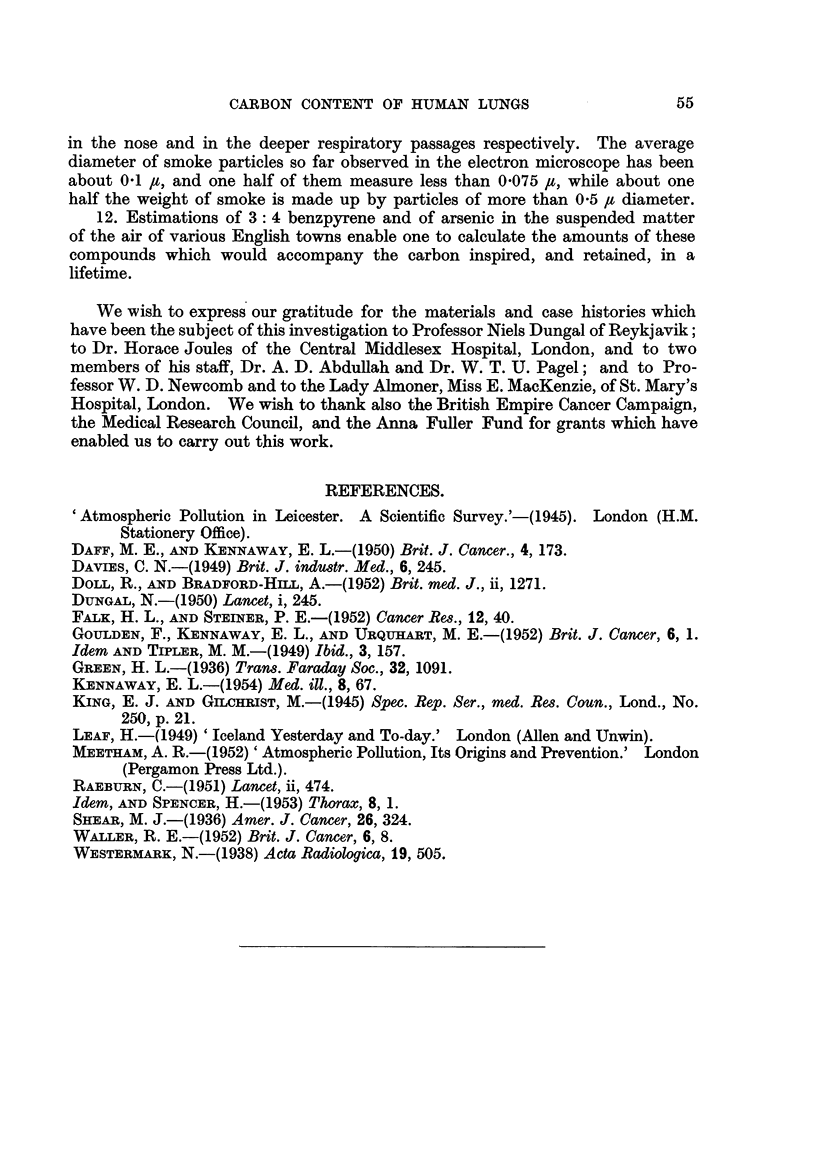# The Carbon Content of Human Lungs and Bronchial Glands*

**DOI:** 10.1038/bjc.1954.4

**Published:** 1954-03

**Authors:** J. W. S. Blacklock, E. L. Kennaway, G. M. Lewis, M. E. Urquhart


### THE CARBON CONTENT OF HUMAN LUNGS AND BRONCHIAL GLANDS.*

J. W. S. BLACKLOCK, E. L. KENNAWAY, G. M. LEWIS AND M. E. URQUHART.

*From the Pathology Department, St. Bartholomew's Hospital, London, E.C.*1.

Received for publication November 28, 1953.

A PORTION of the carbon particles which we inhale is arrested by the mucous membrane of the nose, pharynx and trachea and larger bronchi and in health is expelled by ciliary action. The remainder reaches the lung parenchyma and is nearly always, in the first instance, deposited in the small lymphoid foci around the smaller bronchi and bronchioles. As these become overloaded the excess carbon is carried further afield by lymphatics towards the pleural surface and towards the glands in and around the hilum of the lungs. The tracheo-bronchial glands may occasionally allow small amounts of the carbon to pass through into the thoracic duct or into lymphatic efferents from the glands which communicate directly with the great veins within the thorax, and thus the carbon reaches the blood stream. Such carbon in the blood stream is taken up by the cells of the reticulo-endothelial system in organs like the spleen and liver, which very occasionally at autopsy may show slight stippling with carbon, though in six spleens examined chemically we have found no weighable carbon in our residues.

The amount of carbon deposited in a lifetime in the lungs and tracheo-bronchial glands depends on many variable factors such as age and occupation. Where the degree of atmospheric pollution is great the excessive amount of carbon inhaled clogs the cilia of the respiratory mucous membrane and a large amount is not expelled even in the normal subject. In the subject who suffers from chronic catarrhal inflammation of the respiratory mucosa, there occurs a progressive destruction of the normal ciliated epithelium which is partially replaced by a non-ciliated type of a lower order, such as cuboidal or even squamous cells, which are unable to expel carbon particles. Thus in these subjects, even under the more or less normal conditions of atmospheric pollution that exist in any large town, more carbon will accumulate in the lungs than in the healthy subject. Taking into account such variable factors, the amount of carbon found in the lungs of subjects coming to autopsy must vary within wide limits. Further, the amounts deposited in different parts of the lung vary, as judged by the naked-eye appearance of the lung post mortem. For example, the familiar appearance in the normal lung is that the carbon tends to accumulate mainly under the pleura where the lung lymphatics communicate with those of the pleura through a valved channel which impedes the onward flow of the carbon and also around the hilum in the broncho-pulmonary and tracheo-bronchial lymphatic glands. In

* A preliminary statement of these results was given at a meeting of the European Section of the Union Internationale contre le Cancer at the Institute of Cancer Research, Royal Cancer Hospital, London, on October 19, 1953.

pathological lungs this more or less normal distribution of carbon may be disturbed, due to blocking of lymphatics, as for example by fibrosis around scars resulting from old tuberculosis. The scarring so caused is usually the seat of dense accumulation of carbon. Areas of diffuse fibrosis resulting from bronchiectasis or from the imperfect resolutions of acute pneumonias are often relatively free from carbon. Why this should be so is not altogether clear. Thus the only accurate means of assessing the total amount of carbon in the lungs and tracheo-bronchial glands as a whole is by estimating chemically all the carbon in these structures, and it is from this angle that we have approached the problem. The amount found in these organs at autopsy thus represents what has been retained during a lifetime. The carbon itself may be innocuous, but from our studies of atmospheric pollution (Goulden and Tipler, 1949; Waller, 1952; Goulden, Kennaway and Urquhart, 1952), one can form some idea of the amounts of the carcinogens, 3 : 4 benzpyrene and arsenic, which would accompany it, and may possibly have some effect, either in association with, or apart from, that of tobacco. The black coloration of the lungs and bronchial glands must have become increasingly familiar to British pathologists since the Industrial Revolution which in the earlier part of the nineteenth century converted Britain, by the utilisation of coal and iron, from an agricultural to a largely industrial country.

Although abundant material has been available in Britain during the last 150 years, no one has, so far as we know, attempted to estimate the amount of carbon in human lungs, except in the special case of coal-miners (King and Gilchrist, 1945). When Professor Dungal's paper (1950) on the low incidence of cancer of the lung in Iceland appeared, it was clear that his investigations were yielding very interesting material, and he most kindly sent us the lungs from eight autopsies on persons who had never left Iceland. This material gave unexpected results which will be referred to later.

### MATERIALS.

We wished to study the lungs of people in the following categories: male and female, persons with and without bronchial carcinoma, smokers and non-smokers, town and country dwellers (Table I). Both lungs with the trachea were required in order to obtain the bronchial glands adjacent to the trachea. A questionnaire on the following points was circulated: sex; age; diagnosis and post-mortem findings; places of residence for more than three years and the period lived in each; occupations followed for more than three years; the smoking history as regards the use of a pipe or cigarettes; the age at which smoking was begun and stopped; the maximum and average amounts smoked per day, and whether inhalation was practised always, occasionally or never.

Considerable difficulties were encountered in obtaining lungs from persons without bronchial carcinoma for controls; those were needed from whom an accurate history could be obtained, but who were not likely to live long. We are greatly indebted to Professor W. D. Newcomb of St. Mary's Hospital, London, and to Dr. H. Joules of the Central Middlesex Hospital, London, for the supply of material; also to Professor Neils Dungal of Reykjavik for the lungs of eight persons who had always lived in Iceland. Lungs from six cases were obtained from St. Bartholomew's Hospital, but the histories of these persons were not known.

TABLE I.—*Cases Examined.*

| Male. | | Female. | |
|---|---|---|---|
| *Cases from London.* | | | |
| *Bronchial carcinoma,* 16 *cases* : | | *Bronchial carcinoma,* 5 *cases* : | |
| Smokers | 14 | Smokers | 4 |
| Non-smokers | 2 | Non-smokers | 1 |
| Town dwellers | 16 | Town dwellers | 4 |
| Country „ | 0 | Country „ | 0 |
| | | Not known | 1 |
| *No bronchial carcinoma,* 10 *cases* : | | *No bronchial carcinoma,* 11 *cases* : | |
| Smokers | 4 | Smokers | 2 |
| Non-smokers | 1 | Non-smokers | 9 |
| Not known | 5 | | |
| Town dwellers | 4 | Town dwellers | 9 |
| Country „ | 0 | Country „ | 2 |
| Not known | 6 | | |
| *Total males* | 26 | *Total females* | 16 |
| *Cases from Iceland.* | | | |
| *No bronchial carcinoma,* 4 *cases* : | | *No bronchial carcinoma,* 4 *cases* : | |
| Smokers (all very little) | 3 | Smokers | 0 |
| Non-smokers | 1 | Non-smokers | 4 |

### TECHNIQUE.

1. *Anatomy.*—The tracheo-bronchial and broncho-pulmonary lymph glands were dissected out, the former being sought up to the level of the larynx. The pulmonary glands in the lung substance were separated as far as possible from lung tissue ; no claim is made that this division was carried out very accurately, but the much lower concentration of carbon in the lung makes some contamination with it unimportant. The separation of lymph glands is of course still more difficult when a large invasive mass of bronchial carcinoma is present ; the practice has been to regard as lymph glands any deep black stippled areas of similar size and shape, and to class the remainder as lung. We attach no importance to apparent differences in the distribution of carbon between lung and glands in the various series.

2. *Chemistry.*—The whole lungs were hydrolysed. The estimation of the dry weight of the lung tissue was not attempted because this tissue is not homogeneous, and drying of the whole lung, or its homogenisation so that a representative sample could be taken, is not readily practicable.

The following technique was therefore adopted : The lungs, and bronchial glands, were digested separately by refluxing for 16 hours with an excess of 50 per cent alcoholic KOH (about 350 g. KOH and 700 ml. alcohol to a kg. lung). The clear, dark red supernatant fluid was centrifuged off and the residue washed in 25 per cent HCl, alcohol and acetone until the supernatant fluids were colourless. After drying to constant weight at 110° C. the material was heated to dull red heat and the loss in weight reckoned as carbon. Clearly, estimation of the carbon by direct measurement of $CO_2$ produced was desirable. Samples were sent to Dr. Weiler and Dr. Strauss at the Microanalytical Laboratory, Oxford, for this purpose ; their results amounted consistently to 70 to 80 per cent of our own. It seemed likely that this was due to adsorption of organic materials from the issue hydrolysate which were, like the carbon, combustible at dull red heat. The deposit contained, on average, 1 to 2 per cent of nitrogen and hydrogen. Moreover, it was found that activated charcoal (B.D.H.) added to tissue hydrolysates adsorbed 20 to 30 per cent of its own weight of impurities, whereas sugar charcoal could be quantitatively recovered. The carbon from the lungs resembled the activated charcoal in appearance in that it was much more finely divided than the sugar charcoal. As this investigation was a comparative study, our own figures were used except in a few cases as indicated in the tables.

3. *Histology.*—The lungs submitted to us had been fixed whole in formalin and thus the fixation from a histological point of view was not perfect. Blocks of lung tissue were nearly always taken from the pleural region and from deep in the lung substance, and occasionally from the tracheo-bronchial glands. These blocks were subsequently refixed in formol-corrosive and the paraffin sections were stained by haemotoxylin, by carmalum or neutral red, by van Gieson or Mallory, and, in a certain number of cases referred to later, by potassium ferrocyanide to demonstrate any iron. The sections from each case were studied microscopically for their carbon content, and for the presence of any fibrosis or cancerous infiltration of the lymphatics that might impede free lymphatic drainage of carbon particles. In addition all sections were examined by the polarising microscope to find if there were any doubly refractile granules in the lungs such as silica.

### *The Carbon Content of the Lungs and Bronchial Glands.*

1. *The cases from hospitals in London* (*Tables II and III*).—The places of residence and occupations given are those of longest duration only. In the case of persons living in London, the part of London in which they lived is stated in brackets after a capital L ; for those living in other towns, the county is given in brackets. " Cigarettes " expresses tobacco consumption in this form even where cigars or a pipe were smoked. A cigarette weighs 1 g., hence 1 oz. tobacco is equivalent to 28 cigarettes.

The small number of cases prevents any firm conclusions about differences in the amounts of carbon in the lungs of persons with and without bronchial carcinoma. These differences are not statistically significant, but are nevertheless of interest.

The average amount of carbon in the lungs of females with bronchial carcinoma was greater than that in the controls (Table III), although the carbon content of the glands did not differ appreciably between the two groups of women ; one might expect this result if some carcinogen such as 3 : 4 benzpyrene adsorbed on to the carbon particles in soot is concerned in the aetiology of lung cancer. The two groups of men, however, showed the opposite result. Again, the carbon in the glands was approximately the same for the two groups, but the control group had the higher carbon content in the lungs. There was no apparent reason why men and women should differ in this way.

The total carbon content of a lung must vary according to three chief factors, (*a*) age, (*b*) the size of the lung (since a larger person will inspire more air and hence more carbon), and (*c*) the environment in which a person lived. (*a*) Allowance for the first of these variables is easily made. When the weight of carbon in the lungs and glands was divided by the age (Table IV) the differences between the four groups remained and were still of the same order. The lower carbon content of the lungs of men with bronchial carcinoma is not due to earlier

### TABLE II.—*The Carbon and Ash Content of Human Lungs and Bronchial Glands.*

1. *Males without Bronchial Carcinoma.*

| Ref. No. | Age. | Occupation. | Residence. | Cigarettes. Per day | Cigarettes. Total. | Lungs. g. carbon. | Lungs. g. ash. | Glands. g. carbon. | Glands. g. ash. | Total. g. carbon. | Total. g. ash. | Glands as % total. Carbon. | Glands as % total. Ash. |
|---|---|---|---|---|---|---|---|---|---|---|---|---|---|
| 1 | 77 | Engineer | L. (Harlesden) | 4 | 90,720 | 0·36 | 0·09 | 0·04 | 0·03 | 0·39 | 0·12 | 10·3 | 25·0 |
| 2 | 72 | Bus Conductor | London, N.W.2 | 4 | 78,840 | 1·29 | 0·24 | 0·14 | 0·05 | 1·43 | 0·29 | 9·8 | 17·2 |
| 3 | — | Seaman | W. Indies; L. (Paddington) | 35 | — | 0·89 | 0·23 | 0·14 | 0·05 | 0·94 | 0·28 | 14·9 | 17·8 |
| 4 | 73 | Engineer | Edinburgh; L. (Fulham) | 7 | 341,275 | 1·31 | 0·24 | 0.09 | 0·03 | 1·41 | 0·27 | 6·4 | 11·1 |
| 5 | 57 | — | — | — | — | 0·57 | 0·72 | 0·03 | 0·05 | 0·60 | 0·77 | 5·0 | 6·5 |
| 6 | 60 | — | — | — | — | 0·97 | 0·38 | 0·04 | 0·05 | 1·01 | 0·43 | 4·0 | 11·8 |
| 7 | 30 | — | — | — | — | 0·24 | 0·07 | 0·01 | 0·02 | 0·25 | 0·09 | 4·0 | 22·2 |
| 8 | 84 | Music Teacher | — | 0 | 0 | 0·99 | 0·19 | 0·07 | 0·05 | 1·06 | 0·24 | 6·6 | 20·8 |
| 9 | 70 | Draper | — | — | — | 0·84 | 0·10 | 0·20 | 0·05 | 1·04 | 0·15 | 19·2 | 33·3 |
| 10 | — | — | — | — | — | 0·84 | 0·17 | 0·07 | 0·05 | 0·91 | 0·22 | 7·7 | 22·7 |
| Average | 65 | | | | | 0·83 | 0·24 | 0·08 | 0·04 | 0·90 | 0·28 | 8·8 | 18·8 |

2. *Males with Bronchial Carcinoma.*

| Ref. No. | Age. | Occupation. | Residence. | Cigarettes. Per day | Cigarettes. Total. | Lungs. g. carbon. | Lungs. g. ash. | Glands. g. carbon. | Glands. g. ash. | Total. g. carbon. | Total. g. ash. | Glands as % total. Carbon. | Glands as % total. Ash. |
|---|---|---|---|---|---|---|---|---|---|---|---|---|---|
| 11 | 50 | Meter reader | (L. Harlesden) | 25 | 288,000 | 0·44 | 0·17 | 0·16* | 0·01* | 0·60 | 0·18 | 2·7 | 5·6 |
| 12 | 55 | Decorator | L. (Wembley) | 35 | 441,000 | 0·28 | 0·10 | 0·04 | 0·02 | 0·32 | 0·13 | 12·5 | 15·4 |
| 13 | 67 | Painter | L. (Willesden) | 20 | 345,600 | 0·37 | 0·07 | 0·04 | 0·02 | 0·41 | 0·09 | 9·8 | 22·2 |
| 14 | 65 | Packer | Glasgow; London | 30 | 536,550 | 0·55 | 0·09 | 0·04 | 0·01 | 0·59 | 0·10 | 6·8 | 10·0 |
| 15 | 68 | Bootmaker | L. (Willesden) | 22 | 372,340 | 0·50 | 0·17 | 0·07 | 0·06 | 0·57 | 0·23 | 12·3 | 26·1 |
| 16 | 71 | Commercial traveller | L. (Tooting) | 14 | 282,240 | 0·61 | 0·11 | 0·04 | 0·03 | 0·65 | 0·14 | 6·2 | 14·3 |
| 17 | 43 | Greyhound trainer | L. (Putney); L. (Alperton) | 45 | 443,475 | 0·28 | 0·06 | 0·12* | 0·01* | 0·40 | 0·07 | 30·0 | 14·3 |
| 18 | 52 | Engineer | L. (Fulham) | 35 | 465,900 | 0·60 | 0·08 | 0·02* | 0·01* | 0·62 | 0·09 | 3·2 | 11·1 |
| 19 | 71 | Seaman | Sea, London | 65 | 1,380,600 | 0·70 | 0·15 | 0·06 | 0·03 | 0·76 | 0·18 | 7·9 | 16·7 |
| 20 | 66 | Civil servant | L. (Ealing) | 0 | 0 | 0·87 | 0·25 | 0·06 | 0·05 | 0·93 | 0·30 | 16·5 | 16·7 |
| 21 | 69 | Bus driver | L. (Acton) | 0 | 0 | 0·99 | 0·67 | 0·03 | 0·02 | 1·02 | 0·69 | 2·9 | 2·9 |
| 22 | 63 | None | L. (Harlesden) | 20 | 343,100 | 0·88 | 0·14 | 0·10 | 0·03 | 0·98 | 0·17 | 10·2 | 17·6 |
| 23 | 74 | Tailor | Poland, London | 10 | 211,700 | 1·33 | 0·16 | 0·20 | 0·09 | 1·53 | 0·25 | 13·1 | 36·0 |
| 24 | 68 | Baker | L. (Harlesden) | 30 | 525,600 | 0·99 | 0·14 | 0·08 | 0·06 | 1·07 | 0·20 | 7·5 | 30·0 |
| 25 | 64 | Museums | L. (Willesden) | 25 | 432,000 | 0·57 | 0·16 | 0·05 | 0·03 | 0·62 | 0·19 | 8·1 | 15·3 |
| 26 | 65 | Bootmaker | L. (Kensington) | 15 | 268,275 | 0·89 | 0·14 | 0·04 | 0·02 | 0·93 | 0·16 | 4·3 | 17·0 |
| Average | 63 | | | | | 0·68 | 0·17 | 0·07 | 0·03 | 0·75 | 0·20 | 9·0 | 17·1 |

* Dr. Weiler's estimation.

### TABLE III.—*The Carbon and Ash Content of Human Lungs and Bronchial Glands*—(cont.).

3. *Females without Bronchial Carcinoma.*

| Ref. No. | Age. | Occupation. | Residence. | Cigarettes. Per day. | Cigarettes. Total. | Lungs. g. carbon. | Lungs. g. ash. | Glands. g. carbon. | Glands. g. ash. | Total. g. carbon. | Total. g. ash. | Glands as % total. Carbon. | Glands as % total. Ash. |
|---|---|---|---|---|---|---|---|---|---|---|---|---|---|
| 30 | 75 | Housewife | L. (Acton) | 0 | 0 | 0·34 | 0·12 | 0·04 | 0·04 | 0·38 | 0·16 | 10·5 | 25·0 |
| 31 | 62 | Ladys' maid | S. London | 0 | 0 | 0·50 | 0·18 | 0·17 | 0·07 | 0·66 | 0·25 | 25·8 | 28·0 |
| 32 | 62 | Factory, 3 yr. | Cornwall; L. (Willesden) | 0 | 0 | 0·44 | 0·26 | 0·03 | 0·03 | 0·47 | 0·29 | 6·4 | 10·3 |
| 33 | 50 | Housewife | New Malden (Surrey) | 0 | 0 | 0·13 | 0·05 | 0·02 | 0·05 | 0·15 | 0·09 | 13·3 | 55·6 |
| 34 | 57 | ,, | L. (Acton) | 0 | 0 | 0·29 | 0·08 | 0·11 | 0·06 | 0·40 | 0·14 | 27·5 | 42·9 |
| 35 | 54 | Dress designer | Russia, London | 6 | 52,560 | 0·40 | 0·15 | 0·06 | 0·05 | 0·46 | 0·20 | 13·0 | 25·0 |
| 36 | 52 | P. O. Clerk | Osterley (Mddx.) | 0 | 0 | 0·17 | 0·04 | 0·07 | 0·04 | 0·25 | 0·08 | 28·0 | 50·0 |
| 37 | 33 | Telephone op. | L. (St. Pancras) | 12 | 52,600 | 0·46 | 0·06 | 0·03 | 0·02 | 0·49 | 0·08 | 6·1 | 25·0 |
| 38 | 64 | Housewife | Welling (Kent) | 0 | 0 | 0·16 | 0·04 | 0·03 | 0·02 | 0·19 | 0·06 | 15·3 | 33·3 |
| 39 | 88 | ,, | L. (Wembley) | 0 | 0 | 0·38 | 0·17 | 0·07 | 0·07 | 0·45 | 0·24 | 15·6 | 29·2 |
| 40 | 71 | ,, | Greenford (Mddx.) | 0 | 0 | 0·72 | 0·28 | 0·12 | 0·09 | 0·84 | 0·37 | 14·3 | 24·3 |
| Average | 61 | | | | | 0·36 | 0·13 | 0·07 | 0·06 | 0·43 | 0·18 | 16·0 | 31·7 |

4. *Females with Bronchial Carcinoma.*

| Ref. No. | Age. | Occupation. | Residence. | Cigarettes. Per day. | Cigarettes. Total. | Lungs. g. carbon. | Lungs. g. ash. | Glands. g. carbon. | Glands. g. ash. | Total. g. carbon. | Total. g. ash. | Glands as % total. Carbon. | Glands as % total. Ash. |
|---|---|---|---|---|---|---|---|---|---|---|---|---|---|
| 41 | 60 | School-teacher | L (Fulham); Sudbury (Mddx.) | 8 | 23,040 | 0·24 | 0·18 | 0·07 | 0·03 | 0·31 | 0·21 | 22·6 | 14·3 |
| 42 | 83 | P.O. Clerk | L. (Paddington) | 0 | 0 | 0·47 | 0·21 | 0·05 | 0·06 | 0·52 | 0·27 | 9·6 | 22·2 |
| 43 | 43 | — | Ireland; London, N.W.2 | 35 | 289,800 | 0·45 | 0·12 | 0·02 | 0·06 | 0·47 | 0·18 | 4·3 | 33·3 |
| 44 | 64 | Domestic | L. (Paddington) | 10 | 50,000 | 0·75 | 0·24 | 0·09 | 0·12 | 0·84 | 0·36 | 10·7 | 33·3 |
| 45 | 57 | Cook | — | 40 | 481,800 | 0·95 | 0·41 | 0·06 | 0·06 | 1·01 | 0·47 | 5·9 | 12·8 |
| Average | 61 | | | | | 0·57 | 0·23 | 0·06 | 0·07 | 0·63 | 0·30 | 10·6 | 23·2 |

TABLE IV.—*Total Carbon Content of Lungs with Allowance for Age Differences.*

| | Male. | | | Female. | | |
|---|---|---|---|---|---|---|
| | With bronchial carcinoma. | No bronchial carcinoma. | Difference as percentage. | With bronchial carcinoma. | No bronchial carcinoma. | Difference as percentage. |
| Total g. carbon in lungs and glands (average) . | 0·75 | 0·90 | 17 | 0·63 | 0·43 | 31 |
| Total mg. carbon per year of life (average) . | 11·7 | 13·4 | 12 | 10·6 | 7·4 | 32 |

death since the mean age of this group (63) is almost the same as that of the controls (65). (*b*) No reliable correction for the variation in lung size could be made in the absence of estimations of the dry weight of the lung (see p. 42). (*c*) The effect of environment could not be fully assessed owing to insufficient data. The majority of cases dwelt in London, but over this area the smoke concentration may vary widely. In the group of 11 women without bronchial carcinoma, 2 were entirely country dwellers and had very low values for carbon in the lung, which factor reduces the average for this group from 0·49 to 0·43 g. There is, as one might expect, a relationship between the number of years lived in towns and the carbon content of the lungs (Fig. 1). This graph was constructed

FIG. 1.—The effect of living in towns on the carbon content of human lungs. (Women without bronchial carcinoma.)

from data for women without bronchial carcinoma for two reasons because, first, only in this group were there any real country dwellers, and second, since most of these persons were housewives, their environment was more constant than that of the men ; they would have worked where they lived, whereas the men may have breathed by day the smoky air of a town, and by night the cleaner air of its suburbs.

2. *The cases from Iceland* (*Table V.*)—Since these specimens did not include the whole trachea, some of the bronchial glands may not have been present; the figures in Table V therefore represent the carbon and ash content of the lungs and glands together.

The content of both carbon and ash in the women's lungs was considerably greater than that in the men's, apart from the exceptional value for the man No. 50. The high figures for this case can be explained by the fact that he was a stoker and inhaled large quantities of smoke at work. Comparison of these values with

TABLE V.—*Carbon and Ash Contents of the Lungs from Iceland* (*all without Bronchial Carcinoma*).

| Ref. No. | Age. | Occupation. | Smoking. | Carbon in lungs (g.). | Ash in lungs (g.). | Mg. carbon in lungs/age in years. |
|---|---|---|---|---|---|---|
| | | | 1. *Male.* | | | |
| 50 | 72 | Stoker | Very little | 1·81 | 0·60 | 25·1 |
| 51 | 75 | Clerk | ,, | 0·62 | 0·17 | 8·2 |
| 52 | 75 | — | *Nil* | 0·61 | 0·25 | 8·1 |
| 53 | 65 | Farmer | Very little | 0·59 | 0·15 | 9·1 |
| | | | Average | (0·91)* | (0·29) | (12·6) |
| | | | | 0·60 | 0·19 | 8·5 |
| | | | 2. *Female.* | | | |
| 54 | 76 | — | *Nil* | 1·50 | 0·50 | 13·8 |
| 55 | 74 | — | ,, | 1·14 | 0·18 | 15·4 |
| 56 | 75 | — | ,, | 0·73 | 0·55 | 9·7 |
| 57 | 81 | — | ,, | 0·70 | 2·6† | 8·6 |
| | | | Average | 0·90 | 0·41 | 11·9 |

* Bracketed averages include the abnormal figures for No. 50. See text.
† This very high value for ash was omitted from the average. Microscopically, no silica was found in sections of the lung.

those obtained in London, shows that the lungs of Icelandic men had a lower average carbon content, while those of the Icelandic women had a much larger amount. The same differences obtained on correction of these figures for variation in age distribution.

The presence of such amounts of carbon in these lungs from Iceland seemed at first sight surprising, for in Iceland general atmospheric pollution as understood in this country is very small. There is no native coal or oil, and these are costly to import. The capital city is now heated in part by water from the natural hot springs (Kennaway, 1954). However, in the country, peat is widely used though in some places this is absent, or has been exhausted, and dried sheep's dung is burnt; the confinement of farm stock in winter makes collection easy. Apart from the stoker (No. 50) all these cases lived in the country. Dungal (1950), alluding to the woman No. 57 in Table V, writes that she spent most of her life in the country on a farm. "If she has worked in one of our old-fashioned farm kitchens she will have inhaled a lot of smoke. These kitchens were small rooms with an open hearth, where a fire was kept burning day and night between two stones and the place was frequently full of smoke. There was no chimney. I have seen many of these kitchens in my young days; and as often as not they were full of smoke. In the lungs of this woman, we found microscopically lots of black material, also in a lymphatic node from the neck. The fuel which was burned, and is still chiefly burned in the country, is peat. In spite of all the enormous amounts of peat smoke which must have been inhaled by our womenfolk in their old-time kitchens, cancer of the lung has been practically unknown among them".

Leaf (1949) says that some of the old farmhouses, built of lava blocks with turf between in place of mortar, are now being preserved as national memorials. In one of these at Glaumbaer in Saudarkrokur about 130 miles north of Reykjavik, "the fireplace in the main living room was constructed of brick standing well away from the wall, without a chimney, the smoke escaping as best it could through a hole in the roof". Professor Dungal has procured for us for analysis some sooty material from the walls of one of these farms at Keldur Rangarvallasysla in the south, about 60 miles east of Reykjavik. It will be of interest to compare the benzpyrene content of this material with that of coal soot in England.

These descriptions of the conditions of life on the old farms can well account for the remarkably high carbon content of the women's lungs, and the lower one for men, who would, except in the winter, spend less time indoors.

### SMOKING.

In view of the suggested association between bronchial carcinoma and smoking, especially of cigarettes, the smoking habits of the small number for which adequate data were available were examined, although these are not, of course, statistically significant.

Among men with bronchial carcinoma, 1 out of 8, and of those without bronchial carcinoma, 1 out of 5, was a non-smoker. In the group of women with bronchial carcinoma, 1 out of 5 did not smoke, and in the control group, 9 out of 11. These figures, especially for the women, show a higher proportion of non-smokers among persons without bronchial carcinoma. Dungal (1950) showed that in Iceland consumption of tobacco as cigarettes was very low, and Table V shows that of the cases examined by us, 3 of the 4 men smoked, but all very little, 2 of them being pipe smokers, while none of the 4 women smoked at all.

Doll and Bradford-Hill (1952) found that pipe smoking was less associated with bronchial carcinoma than cigarette smoking, possibly because the pipe-stem acts as a filter. In the small group studied here, the only 2 men who were " pure " pipe smokers were persons without bronchial carcinoma. In the group of men with bronchial carcinoma, none smoked a pipe only, 1 a pipe and cigars, 1 a pipe and cigarettes, 3 rolled their own cigarettes and 9 smoked manufactured cigarettes. Among 4 men without bronchial carcinoma, 2 smoked a pipe only and 2 smoked manufactured cigarettes. In the cases examined here, 8 out of 11 smokers were reported as having always inhaled.

There appears to be no relationship between the number of cigarettes smoked in a lifetime and the amount of carbon accumulated in the lungs and bronchial glands (Fig. 2). The total carbon content of the lungs varied over a wide range for non-smokers ; all the points indicating a total consumption of more than 200,000 cigarettes refer to men and women with bronchial carcinoma. The average daily consumptions were : males with bronchial carcinoma, 24/day (16 cases) ; males without bronchial carcinoma, 12/day (5 cases) ; females with bronchial carcinoma, 19/day (5 cases); and females without bronchial carcinoma, 2/day (10 cases).

*Ash Content of the Lungs and Bronchial Glands.*

The nature of the ash deposited from the hydrolysates of these lungs has not been determined. Although about 10 per cent of the total carbon was contained in the glands, the fraction of the total ash present in them was about 20 per cent. This suggested that the bronchial glands were even more efficient in concentrating particles of mineral matter than they were in accumulating carbon particles. The

Fig. 2.—Cigarette smoking and the carbon content of human lungs.

× . . . . . . ♀ with bronchial carcinoma.

● . . . . . . ♀ without ,, ,,

○ . . . . . . ♂ with ,, ,,

▲ . . . . . . ♂ without ,, ,, absolute values for ash in the lungs were very nearly the same in men and in women, but ash formed a higher proportion of the total deposit in the lungs of women; namely, 23·9 per cent and 29·6 per cent of the total deposit for women without and with bronchial carcinoma, as against the corresponding figures for men, 18·0 per cent and 19·3 per cent. It seems possible that this may be due to the housewifely duty of cleaning grates, when a considerable quantity of ash might be inhaled.

*Microscopical Examination of Tissues.*

For a true histological estimation of the carbon present in the lungs, sections of the whole lung are essential, but in view of the chemical estimations required in this work, whole sections were not possible. As already indicated, only two blocks of tissue were taken from the lungs for microscopic study, which showed a wide variation in the amount of carbon. For purposes of record, these variations were classified as (*a*) none or slight, (*b*) moderate, and (*c*) fairly marked. No dense diffuse carbon depositions such as are seen in the lungs of coal-miners or coal-trimmers were observed. For comparison with the histological findings the chemical results were divided into three groups, namely, those with a carbon content of (*a*) 0·49 g. or less, (*b*) 0·4 to 0·7 g., and (*c*) 0·8 g. and over. In general there was a fairly close agreement between the histological and chemical results in these categories. In class (*a*) the carbon content was more in the histological section than the chemical results indicated in 2 out of 18 cases. In both cases this divergence could be accounted for by localised areas of dense fibrosis which were heavily laden with carbon. In class (*b*) the results were in agreement in 11 of the 15 cases. In the other 4, very diffuse cancerous infiltration of the lymphatics was present and in these the carbon in the sections was less than was expected from the chemical result. In class (*c*) the results agreed in 14 of the 17 cases ; in the remaining 3 cases the histological evidence showed less carbon than was expected from the chemical results and in these cases the lymphatics were heavily infiltrated with cancer cells. The carbon in the sections showed the normal distribution, being heavier under the pleura, around the bronchi and blood vessels and in the fibrous septa, than in the vesicular portions of the lung.

The rocks of Iceland are of volcanic origin and the roads are metalled with such material which, due to weathering, quickly breaks down so that with the passing of traffic much dust is created. Probably road dust has become considerable in Iceland, where there are now 11,000 motor vehicles, only since the acquisition of American cars after the last war. In view of this the Icelandic lungs were submitted to a more intense microscopic study for the presence of silica and of iron. No evidence of silicosis was noted in any and doubly refractile granules of silica were very scanty or absent in all the sections. In only one case was iron found, apart from occasional heart-failure cells in the others. In this case the iron was present chiefly in the fibrous tissue of the septa and around the bronchi and blood-vessels in close association with carbon particles which the iron pigment appeared to surround. The case was a woman, aged 75, with a carbon content of 0·73 g. and an ash of 0·55 g. For comparison with the Icelandic cases, 8 lungs were studied for iron from subjects in the British series. The British cases corresponded in age and sex to the Icelandic but, apart from some heart-failure cells, no iron was found in any case.

### *Atmospheric Pollution.*

In considering the amounts of carbon found in these lungs, one is led to calculate the quantity of carbon in the form of smoke likely to be inhaled in a lifetime, and the fraction of it that is retained in the lung.

Some short term is required to designate the suspended matter in the air of a town, and " smoke", to which various objections may be raised, is generally used. The Leicester Report (1945) says (p. 8) " The word ' smoke ' will be used throughout this report for the black suspended impurities collected and indirectly weighed by air filters. It is shown that the principal constituent of suspended matter is soot, so that ' smoke ' describes it better than ' dust ' ".

*Deposited and Suspended Matter in the Centre of Leicester during October*, 1938.

| | Per cent by weight. Suspended matter. | Deposited matter. |
|---|---|---|
| Tar (soluble in $CS_2$) . . | 14 | 1 |
| Other combustible matter . | 71 | 30 |
| Ash . . . . . | 15 | 70 |

The composition of the " smoke " of Leicester is shown above.

A very comprehensive study of atmospheric pollution in Leicester (1945) was carried out during 1937–38 under the direction of the Atmospheric Pollution Research Committee of the Department of Scientific and Industrial Research. This town was selected because of its medium size (population 260,000 ; diameter as a source of smoke, from 3 to 6 miles), its approximately circular outline, the unusual isolation from other large cities, and the location of almost all the factory chimneys within a mile from the city centre. Table VI summarises the data obtained.

TABLE VI.—*Mean Concentration of Smoke, Leicester*, 1937–39. (The stations 1–4 are those nearest to the centre of the town.)

| | Smoke mg. /100 m³.. | | | | | |
|---|---|---|---|---|---|---|
| Stations . | 1. | 2. | 3. | 4. | Mean. | |
| Summer (May–Sept.), 1937 . . | 16 | 13 | 12 | 12 | 13 | 13 |
| " " 1938 . . | 18 | 12 | 11 | 12 | 13 | |
| Winter (Jan.–Mar.), 1937 . . | 38 | 20 | 25 | 18 | 25 | 30 |
| Winter (Nov.–Mar.), 1937–38 . . | 46 | 33 | 36 | 29 | 36 | |
| " " 1938–39 . . | 38 | 26 | 27 | 24 | 29 | |
| Whole year . . . . . . | — | — | — | — | 21·5 | |

Elsewhere in the report on Leicester (p. 123), a rather higher range of figures is given, of which the mean for the whole year would be 29 mg./100 m³. " In summer the mean smoke concentration at the centre of Leicester was 170 $\mu$g./m³. The highest daily mean was 3·7 times as much. The highest hourly mean was 7·1 times as much. In winter the corresponding concentrations were 410 for the mean, 4·4 times as much for the highest daily mean, and 5·4 times as much for the highest hourly mean." The only other data of this kind which we have found are those of Meetham (1952, p. 134) who gives the following figures for suspended impurities " at street level in British towns " and in the country (Table VII).

TABLE VII.—*Suspended Impurities* (Meetham, 1952).

| | Smoke mg. /m.³. On a hazy winter day. Town. | Country. | In summer. Town. | Country. |
|---|---|---|---|---|
| Combustible solids and liquid particles . . | 1·0 | 0·24 | 0·2 | 0·05 |
| Incombustible solids . | 0·2 | | | |

*The Amount of Carbon Inspired in a Lifetime.**

The " Standard Man " inspires about 20 m.$^3$ of air per day, which is 511,000 m.$^3$ in a lifetime of 70 years. This volume is about 4 times that of the Albert Hall in London. If this air contained the average amount of smoke found in Leicester (21·5 mg. in 100 m.$^3$), the total amount inspired would be 110 g. From the figures for the carbon content of the lungs examined (Tables II, III, IV) it is seen that only 0·25 to 1·0 per cent of this 110 g. is retained ; this gives some measure of the efficiency of the nasal filter and of the mechanism by which particles which have reached the deeper respiratory passages are swept upwards again by ciliary action and expectorated or swallowed. The most common site for cancer of the lung is stated to be the two main bronchi and their largest branches. When the floor of a room is swept towards the door, the part adjacent to the door is exposed to dirt from a much larger area. Similarly, when particles of dust are arrested on the walls of the smaller bronchi and bronchioles, they are swept upwards by ciliary action through the main bronchi (the door) into the trachea, and carcinogens might diffuse from the particles into the layer of mucus covering the bronchial mucous membrane during transit. Some such mechanism would be in accord with the statement about the site quoted above ; but some recent investigators (Westermark, 1938 ; Raeburn, 1951 ; Raeburn and Spencer, 1953) think that tumours arise with about equal frequency in any part of the lung.

The estimations of arsenic and of 3 : 4 benzpyrene in the air of large towns given in Table VIII are those of Goulden, Kennaway and Urquhart (1952) and

TABLE VIII.—*Concentrations of Arsenic and of* 3 : 4 *Benzpyrene in Town Air.*

Mean concentrations, $\mu$g. /100 m.$^3$ of air.

| | Arsenic ($As_2O_3$). | | 3 : 4 Benzpyrene. |
|---|---|---|---|
| London (Becton) | 13·2 | London (Becton) | 7·1 |
| Bilston | 7·5 | London (County Hall) | 4·6 |
| Manchester | 7·3 | Sheffield | 4·2 |
| Liverpool | 6·8 | Leicester | 2·9 |
| Sheffield | 6·5 | Bilston | 2·7 |
| London (County Hall) | 5·8 | Burnley | 2·7 |
| Hull | 5·4 | Cannock | 1·9 |
| Bristol | 3·7 | Hull | 1·8 |
| | | London (Crossness) | 1·6 |
| | | Bristol | 1·3 |
| Mean | 7·0 | Mean | 3·1 |

Waller (1952). We do not know of any corresponding figures for purely rural areas. These mean concentrations (7·0 $\mu$g. arsenic ($As_2O_3$) and 3·1 $\mu$g. 3 : 4 benzpyrene in 100 m.$^3$) would give 36 mg. arsenic and 16 mg. 3 : 4 benzpyrene in the 511,000 m.$^3$ breathed in a lifetime of 70 years. This amount of arsenic would be contained in 7 maximum doses of the Fowler's solution of the British Pharmacopoeia (1 per cent $As_2O_3$) or in 720 cigarettes of the brands most commonly smoked in this country which contain an average of 50 $\mu$g. $As_2O_3$ per cigarette (Daff and Kennaway, 1950). Shear (1936) found that the minimum amount of a strong carcinogen such as 1 : 2 : 5 : 6 dibenzanthracene which will produce

* Our earlier results, which were confirmed subsequently, were demonstrated at a meeting of the Pathology Section of the Royal Society of Medicine held at this hospital on January 15, 1952.

sarcoma subcutaneously in a mouse is less than 0·4 $\mu$g., an amount contained 40,000 times in the 16 mg. of 3 : 4 benzpyrene which may be inspired in a lifetime, but in view of the vastly greater area over which this may be spread and the different time relations, one may doubt whether this comparison has any value.

*The Size and Arrest of Smoke Particles.*

The available data about the arrest of smoke particles in the air passages are very defective because we have not, to begin with, any adequate information about the size of these particles. The passages quoted verbatim below contain all the information that we have found on this subject.

The Leicester Report says: " The size distribution of smoke is a subject which still requires investigation. Green (1936) gives estimates for five industrial dusts from which it can be inferred that 50 per cent by weight of such dusts is less than 3 to 10 microns, and 80 per cent is less than 5 to 15 microns in mean diameter. Atmospheric smoke may be similar in size distribution but a direct investigation is still needed . . . The smoke filter therefore is an efficient instrument for sampling suspended matter in the air of Leicester because there are not enough particles of sizes greater than 10 microns to matter . . . " (p. 14). " Smoke particles appear to have an average diameter of about 0·1 micron " (p. 71). Electron microphotographs of smoke showed (Meetham, 1952, p. 178) that " when records containing about 100,000 particles were examined, it was found that half the individual smoke particles were smaller than about 0·075 micron ;* but half the weight of smoke was in particles larger than about 0·57 micron. Some of the properties of smoke depend on the area it can cover ; half the surface or sectional area of the smoke was in particles larger than about 0·35 micron. There is a distinct tendency for smoke particles to stick together in chains, perhaps one micron long."

The comparison of such results obtained by different methods of arrest is not easy. Dr. E. T. Wilkins of the Fuel Research Station in London (personal communication), says " There are, of course, certain difficulties in obtaining a particle size analysis of a filter-paper deposit and the only record we have relates to a sample collected by means of a thermal precipitator at Teddington in 1943. The particles which comprise this sample, when examined with an electron microscope, appeared to contain about 17 per cent by weight of particles greater than 0·4 $\mu$, but I am not satisfied that this analysis could be considered typical". The practical significance of the word " average " in relation to particle size is hard to define.

Table IX comprises 2 sections :

(*a*) Data for the arrest of particles in the respiratory tract taken from those collected by Davies (1949) ; it is not always clear whether such data rest on a deductive or experimental basis. Smoke particles having an average diameter of 0·1 $\mu$. are in the range of size where there is minimum retention in the lung, 80 per cent of them being exhaled and 20 per cent arrested in the alveoli.

(*b*) The mean diameter of smoke particles and the range of particle size of carbon black which shows the greatest adsorption of 3 : 4 benzpyrene from benzene (Falk and Steiner, 1952). Under certain conditions the hydrocarbon could not be eluted from particles 10–17 m$\mu$. diameter ; we do not, of course, know if the

* This is 1/100 of the average diameter of a human red blood corpuscle (Fig. 3).

TABLE IX.—*The Arrest of Particles in the Respiratory Tract.*

| Radius of particle. | Mechanism of arrest. | Site of arrest. Nose (per cent). | Bronchi (per cent). | Bronchioles. | Alveoli. | Percentages exhaled. | Remarks. |
|---|---|---|---|---|---|---|---|
| (a) *Smoke* | | | | | | | |
| 5 μ and above | Impingement | 80 | 20 | 0 | 0 | 0 | — |
| 2 μ | Sedimentation | 50 | Small | Considerable | Small | 0 | Maximum in bronchi. |
| 0·9 μ | — | 20 | — | — | — | 50 | — |
| 0·4 to 0·8 μ | Sedimentation | 20 | — | — | — | — | Maximum in alveoli and bronchioles. |
| 0·3 μ | Brownian movement | — | — | — | — | — | — |
| 0·1–0·15 μ | ,, | 0 | 0 | 0 | 20 | 80 | Minimum retention. |
| Less than 0·1 μ | ,, | 0 | 0 | Small | Considerable. | — | Arrest by Brownian movement increases. |
| (b) 0·1–0·075 μ | Average diameter of smoke particle (see p. 52) | | | | | | |
| *Carbon Black* | | | | | | | |
| 0·01–0·017 μ | Complete adsorption of 3 : 4 benzpyrene under certain conditions (Falk and Steiner, 1952). | | | | | | |

compound thus adsorbed would be carcinogenic. The average smoke particle is ten times this diameter.

The sizes of some of the particles mentioned in this section are shown in Fig. 3.

FIG. 3.—Size of particles. Carbon black indicates particle within range (10 to 17 mμ.) giving maximum adsorption of 3 : 4 benzpyrene.

SUMMARY.

1. Estimations of carbon have been carried out on the lungs and bronchial glands of 50 persons. This number is, of course, statistically inadequate, but the results are brought forward now in view of the difficulty in obtaining suitable material.

2. This material was obtained from hospitals in London (42 cases) and in Iceland (8 cases).

3. The organs were hydrolysed by alcoholic KOH. The solid residue, separated by centrifugation, was washed with various solvents, dried, weighed and combusted. The material lost on combustion was reckoned as carbon and the remainder as ash. This " carbon " yielded about 70 to 80 per cent of the calculated amount of $CO_2$. Carbon, in the form of sugar charcoal, added to such mixtures was quantitatively recovered by this technique.

4. Various categories, *i.e.*, men and women ; persons with and without bronchial carcinoma ; smokers and non-smokers ; residents in town and country, were compared as far as the data allowed, and the occupations noted.

5. The mean total amount of carbon in the lungs and bronchial glands is, in the material from hospitals in London, higher in men than in women. The cases of bronchial carcinoma show, in comparison with controls, smaller amounts in men and larger amounts in women. About 10 per cent of the total carbon was contained in the lymph glands. The material from Iceland, which included no cases of bronchial carcinoma, showed the reverse relation between men and women; this difference might be explained by the conditions of rural life in that country in the recent past.

6. Though the material was not statistically adequate, the smoking histories of the cases examined showed a higher consumption of tobacco among both men and women with bronchial carcinoma compared with the controls. The amount of carbon in the lungs showed no relation to tobacco consumption.

7. A relationship was apparent between the accumulation of carbon in the lungs and the duration of life in London or other large towns.

8. The histological distribution and abundance of carbon particles was compared with the results of the chemical analyses.

9. If the carbon in the lungs and bronchial glands does not leave the body by any channel the amount found post-mortem represents what is retained out of the intake of a lifetime. The data now available for atmospheric pollution in some English towns allows an estimate to be made of the amount inspired during a lifetime in them ; this might be of the order of 100 g., while the quantity found in the lungs and bronchial glands was approximately 0·5 to 1·0 g. The difference between these two amounts is a measure of the efficiency of the mechanisms in the nasal and tracheo-bronchial mucous membranes for the arrest and expulsion of particles.

10. Statements collected from the literature indicate that particles of more than $5\mu$ diameter are stopped more or less completely in the nose ; smaller ones penetrate into and are arrested in the respiratory passages to varying degrees according to size.

11. The data available on the size distribution of smoke particles in the air of towns are very scanty indeed, and one cannot with much success apply the data given under (9) above to decide the proportions of such particles arrested in the nose and in the deeper respiratory passages respectively. The average diameter of smoke particles so far observed in the electron microscope has been about 0·1 $\mu$, and one half of them measure less than 0·075 $\mu$, while about one half the weight of smoke is made up by particles of more than 0·5 $\mu$ diameter.

12. Estimations of 3 : 4 benzpyrene and of arsenic in the suspended matter of the air of various English towns enable one to calculate the amounts of these compounds which would accompany the carbon inspired, and retained, in a lifetime.

We wish to express our gratitude for the materials and case histories which have been the subject of this investigation to Professor Niels Dungal of Reykjavik; to Dr. Horace Joules of the Central Middlesex Hospital, London, and to two members of his staff, Dr. A. D. Abdullah and Dr. W. T. U. Pagel; and to Professor W. D. Newcomb and to the Lady Almoner, Miss E. MacKenzie, of St. Mary's Hospital, London. We wish to thank also the British Empire Cancer Campaign, the Medical Research Council, and the Anna Fuller Fund for grants which have enabled us to carry out this work.

### REFERENCES.

'Atmospheric Pollution in Leicester. A Scientific Survey.'—(1945). London (H.M. Stationery Office).

DAFF, M. E., AND KENNAWAY, E. L.—(1950) *Brit. J. Cancer.*, **4**, 173.

DAVIES, C. N.—(1949) *Brit. J. industr. Med.*, **6**, 245.

DOLL, R., AND BRADFORD-HILL, A.—(1952) *Brit. med. J.*, ii, 1271.

DUNGAL, N.—(1950) *Lancet*, i, 245.

FALK, H. L., AND STEINER, P. E.—(1952) *Cancer Res.*, **12**, 40.

GOULDEN, F., KENNAWAY, E. L., AND URQUHART, M. E.—(1952) *Brit. J. Cancer*, **6**, 1.

*Idem* AND TIPLER, M. M.—(1949) *Ibid.*, **3**, 157.

GREEN, H. L.—(1936) *Trans. Faraday Soc.*, **32**, 1091.

KENNAWAY, E. L.—(1954) *Med. ill.*, **8**, 67.

KING, E. J. AND GILCHRIST, M.—(1945) *Spec. Rep. Ser., med. Res. Coun.*, Lond., No. 250, p. 21.

LEAF, H.—(1949) 'Iceland Yesterday and To-day.' London (Allen and Unwin).

MEETHAM, A. R.—(1952) 'Atmospheric Pollution, Its Origins and Prevention.' London (Pergamon Press Ltd.).

RAEBURN, C.—(1951) *Lancet*, ii, 474.

*Idem*, AND SPENCER, H.—(1953) *Thorax*, **8**, 1.

SHEAR, M. J.—(1936) *Amer. J. Cancer*, **26**, 324.

WALLER, R. E.—(1952) *Brit. J. Cancer*, **6**, 8.

WESTERMARK, N.—(1938) *Acta Radiologica*, **19**, 505.